# Stable, interactive modulation of neuronal oscillations produced through brain-machine equilibrium

**DOI:** 10.1016/j.celrep.2022.111616

**Published:** 2022-11-08

**Authors:** Colin G. McNamara, Max Rothwell, Andrew Sharott

**Affiliations:** 1 MRC Brain Network Dynamics Unit, Nuffield Department of Clinical Neurosciences, University of Oxford, Oxford OX1 3TH, UK; 2 Lead contact

## Abstract

Closed-loop interaction has the potential to regulate ongoing brain activity by continuously binding an external stimulation to specific dynamics of a neural circuit. Achieving interactive modulation requires a stable brain-machine feedback loop. Here, we demonstrate that it is possible to maintain oscillatory brain activity in a desired state by delivering stimulation accurately aligned with the timing of each cycle. We develop a fast algorithm that responds on a cycle-by-cycle basis to stimulate basal ganglia nuclei at predetermined phases of successive cortical beta cycles in parkinsonian rats. Using this approach, an equilibrium emerges between the modified brain signal and feedback-dependent stimulation pattern, leading to sustained amplification or suppression of the oscillation depending on the phase targeted. Beta amplification slows movement speed by biasing the animal’s mode of locomotion. Together, these findings show that highly responsive, phase-dependent stimulation can achieve a stable brain-machine interaction that leads to robust modulation of ongoing behavior.

## Graphical abstract

**Graphic abstract:**
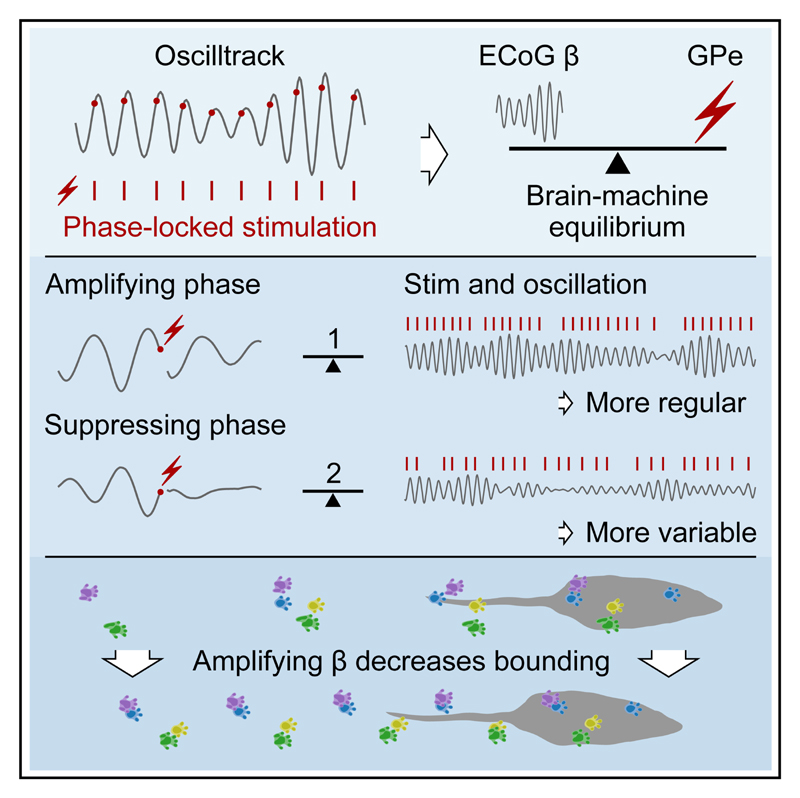

## Introduction

Oscillatory activity in the brain is supported by reciprocal connectivity that allows the firing of action potentials in connected areas to be precisely timed in relation to each other at specific frequencies (Buzsáki and Draguhn, 2004). When recorded in the local field potential or electrocorticogram (ECoG), such activities typically wax and wane in amplitude, indicating the fluctuating strength and phase stability of these oscillatory interactions overtime (Buzsáki et al., 2012; Feingold et al., 2015). Importantly, single oscillatory cycles are proposed to provide a temporal framework to bind functionally related activity in many brain networks (Buzsáki and Draguhn, 2004; Fries, 2005; Lisman and Jensen, 2013). Achieving precise manipulation of neuronal oscillations is of primary importance given their ubiquitous association with widespread brain functions (Buzsáki et al., 2012) and role in many prominent brain disorders including schizophrenia, depression, motor disorders, Alzheimer’s disease, addiction, and epilepsy (Schnitzler and Gross, 2005; Uhlhaas and Singer, 2006). Manipulating oscillations with individual cycle accuracy—and thus with computationally relevant timing—has the potential to provide restoration of dysfunctional systems.

The functional timescales of fluctuating neuronal oscillations, however, are challenging to target precisely using conventional methods and require near-millisecond precision. Suppression of oscillations can be reliably achieved by disrupting a network node involved in their generation, but this will heavily affect many unrelated processes and does not allow bidirectional manipulation. An alternative approach is to deliver a perturbation on a specific phase of the oscillation (Busch et al., 2022; Cagnan et al., 2019a; McNamara and Sharott, 2020; Pikovsky et al., 2003; Siegle and Wilson, 2014; Wodeyar et al., 2021; Zanos et al., 2018), which can energize or dampen ongoing activity (Brittain et al., 2013; Cagnan et al., 2017; Escobar Sanabria et al., 2020; Holt et al., 2019; Kanta et al., 2019; Nicholson et al., 2018; Peles et al., 2020; Rosin et al., 2011; Schreglmann et al., 2021; Takeuchi et al., 2021). Hitherto, these approaches have been defined by the effect of the perturbation on the amplitude or phase of input oscillation. Alternatively, if the parameters of the closed loop were to allow a rapid response to these perturbations, a bidirectional interaction could develop between the signal and the timing of stimulation. Such a fast-acting system, continuously pushing a brain network toward a desired state, has wide-ranging potential to provide physiologically relevant manipulations. The interaction should be sufficiently light touch that the brain network is still free to exhibit relevant physiological fluctuations. This interactive modulation would be fundamentally different and complementary to closed-loop approaches that drive conventional stimulation in response to neural activity associated with a specific behavioral and/or cognitive state (Basu et al., 2021). Such existing approaches are reactive, responding to behaviorally relevant activity with the aim of influencing it as it arises. In contrast, holding a neuronal oscillation in a given state through a fast timescale interaction has the potential to regulate network activity and, in turn, deterministically bias the network’s influence on behavior. This is attractive for therapeutic intervention as it could be used to maintain a network in a more functional state rather than intermittently responding to epochs of pathological activity. However, the feasibility and potential of this type of brain-machine interaction remains to be established. It requires a stable feedback loop where the combined brain-machine system enters into a broad state of equilibrium. Here, we sought to establish and characterize such a feedback loop.

Many state-of-the-art developments in closed-loop stimulation have been in the treatment of Parkinson’s disease (PD), where high-frequency deep brain stimulation (DBS) of basal ganglia structures is an effective treatment. Abnormally powerful beta oscillations in basal ganglia networks are a well-established biomarker of akinetic rigid symptoms (Kühn et al., 2006; Sharott et al., 2014). “Adaptive” DBS, whereby high-frequency stimulation is triggered reactively on the detection of “bursts” of beta activity, produces equivalent symptom amelioration to continuous stimulation (Little et al., 2013; Tinkhauser et al., 2017). The parkinsonian brain thus provides a highly relevant platform to test the potential of interactive modulation in regulating disease-related activity. Here, in PD model rats, we demonstrate that a stable brain-machine equilibrium can emerge from closed-loop stimulation delivered to the basal ganglia at specific phases of a cortical oscillation. By changing the target phase, we could alter the point of equilibrium to suppress or amplify the beta oscillation, with consequent changes in ongoing behavior. Use of a rodent model allowed us to show that the amplifying and suppressing phases and their behavioral effects were highly consistent across many animals. These findings provide proof-of-principle evidence that interactive modulation of neuronal oscillations—where interventions are accurately aligned with the timing of successive individual cycles—is a viable approach for reliably biasing behavior associated with dysfunctional brain networks.

## Results

### Real-time phase tracking and phase-locked stimulation

To enable the sustained application of phase-dependent stimulation, we developed an approach for continuous real-time phase estimation with zero filter delay (McNamara and Sharott, 2020), which we implemented as a digital circuit using the existing hardware of a commercially available recording system. Ordinarily, to produce such an online phase estimate, the signal would be filtered with a pass band filter before a phase estimation step such as the Hilbert transform or the detection of zero crossings (Brittain et al., 2013; Cagnan et al., 2017; Escobar Sanabria et al., 2020; Kanta et al., 2019; Peles et al., 2020; Schreglmann et al., 2021; Siegle and Wilson, 2014; Takeuchi et al., 2021; Zanos et al., 2018). Such filters exhibit a filter delay, whereby the estimate at a given time point pertains to a fixed time in the past. While easily corrected in offline analysis, when calculating in real time, this results in a delay that typically can amount to half a cycle or more. This delay (which is due to the algorithm, not the hardware on which it is implemented) coupled with loss of signal around stimulation artefacts and further delays due to implementation hardware represent challenges in realizing a system that can react responsively on a cycle-by-cycle basis. Our algorithm, which we named OscillTrack, operates directly on the wideband signal and provides a phase estimate for each sample with zero filter delay (see STAR Methods). Here, implementation as a digital circuit in the low-level hardware of the recording system ensured that associated delays were insignificant. Together, these innovations enabled the continuous delivery of phase-targeted stimulation with each stimulus informed by the immediately preceding cycle. No additional hardware was required beyond a means of TTL-triggered stimulation and a recording system with the ability to add customizations to a field-programmable gate array (FPGA) handling the data stream such as those available from Intan Technologies. Electrical stimulation was used here, but the approach is equally suited for use with optogenetic stimulation. We also developed and optimized the algorithm for use in embedded settings such as low-power implantable devices, and we provide microcontroller code to facilitate its use in a wide range of settings (see https://colinmcn.github.io/OscillTrack/).

**Figure 1:**
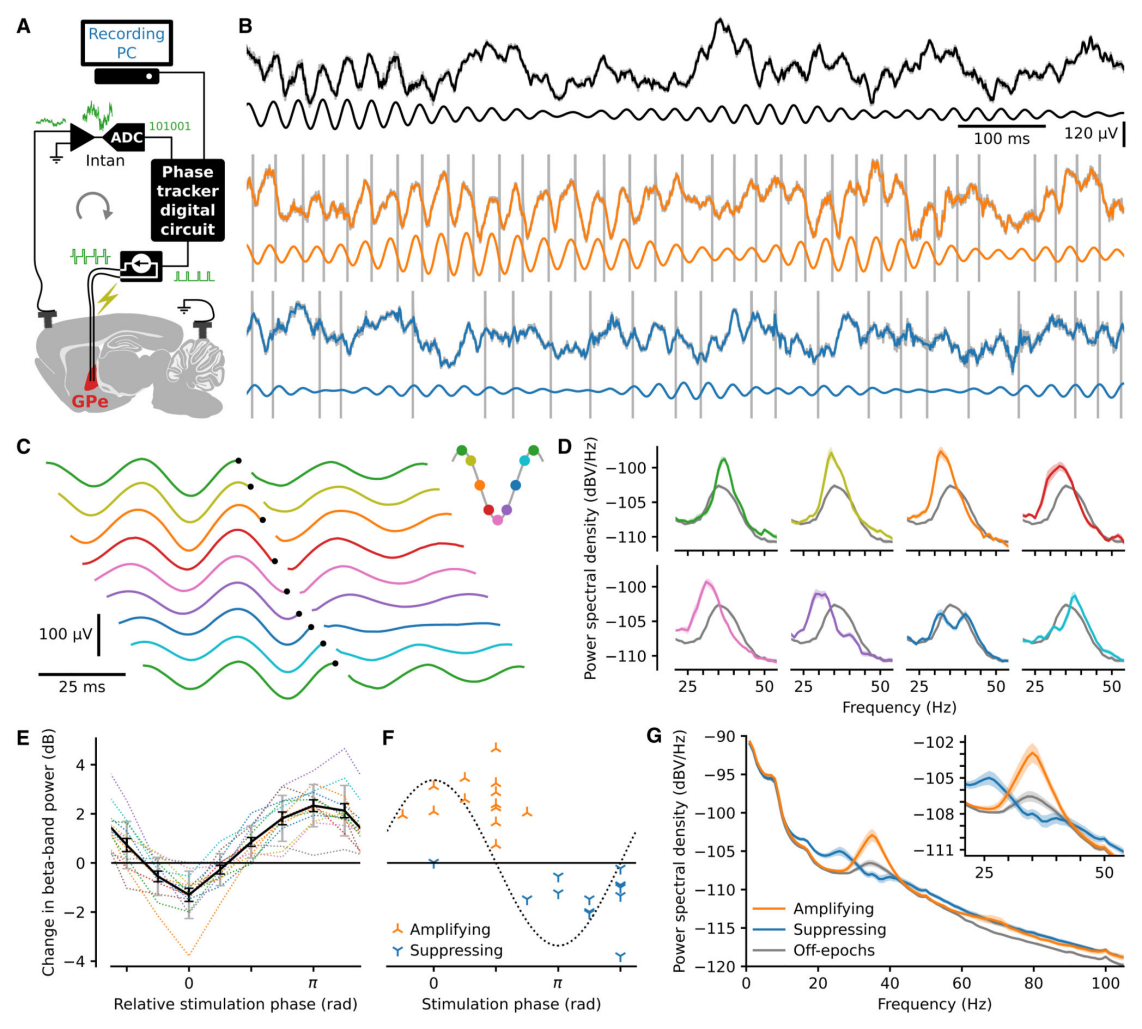
Phase-dependent modulation of beta-frequency oscillations (A) A digital circuit triggered delivery of electrical pulses to the globus pallidus at a predetermined beta-band ECoG phase. (B) Example 1 s of raw wideband EcoG traces (pre-artefact removal) in awake, freely behaving rats (gray). Traces with artefact removal and downsampling (calculated post hoc) are superimposed (black, orange, and blue). The superimposed traces largely cover the original raw trace except at the time of the artefacts (grey). The artefacts are truncated in amplitude due to the limited plot area. The artefact-removed trace, band-pass filtered in the beta-band, isshown below each trace. Three conditions are shown: no stimulation (top, black), stimulation targeted to mid-descending phase producing amplification (middle, orange), and targeted to the mid-ascending phase producing suppression (bottom, blue). (C) Stimulation triggered averages from the same example block of EcoG recordings, targeted to eight equally spaced phases. To aid visualization, triggertimes (black dots) are staggered across traces, and the first trace is repeated. (D) Power spectra (mean ± SEM) from stimulation on-epochs for each target phase (colors as in C) from the same example recordings and for all off-epochs imbedded in those same recordings (gray). (E) Change in beta-band power (on-compared with off-epochs) due to stimulation across 13 rats for 8 target phases aligned by the most suppressing target phase for each rat. Dotted lines show the values for each rat with mean ± SEM in black and SD in gray. (F) Absolute phase versus change in beta-band power of the most suppressing and most amplifying target phases for each rat. The dotted line represents the beta cycle. (G) Power spectral density plots (mean ± SEM, n = 13 rats).

To demonstrate the utility of our approach, we performed phase-locked stimulation of the pathologically exaggerated beta oscillations (Figures 1A and S1) that occur in the parkinsonian cortico-basal ganglia network (Hammond et al., 2007). Electrical stimulation was delivered to the globus pallidus (GPe) of 6-hydroxydopamine (6-OHDA) hemi-lesioned rats at 8 equally spaced target phases of the ongoing ECoG beta oscillation. The GPe is a key node in the generation of parkinsonian beta oscillations (Crompe et al., 2020; Mallet et al., 2008). As previously reported (Avila et al., 2010; Brazhnik et al., 2014), the peak frequency in these rats was in the high beta range between 35 and 40 Hz. While this range would often be described as low gamma in other circuits (Buzsáki and Draguhn, 2004), we will label it beta frequency in line with previous studies in this field. Stimulation consisted of a pair of charge-balanced consecutive pulses of opposite polarity, each 50, 60, or 70 μA in amplitude and 95 μs in duration, delivered across an electrically isolated pair of adjacent stainless-steel electrodes. The real-time system generated triggers during 20 s epochs (on-epochs) separated by 5s trigger-free epochs (off-epochs). Across all phases, 58.66% ± 4.71% (mean ± SD, n = 13 rats) of the stimuli were delivered within a quarter of a cycle of the target phase. This distribution was almost identical to that produced by running the algorithm in real time with the stimulation disabled and thus without stimulation artefacts (p = 0.093, Kolmogorov-Smirnov [KS] test, KS = 0.22; Figure S1C), suggesting that phase-tracking accuracy did not degrade with the addition of stimulation artefacts.

### Phase-dependent modulation of oscillatory power

It was apparent in the raw data that stimulation at some target phases reduced beta-band activity, whereas others increased it (Figures 1B–1D). Absolute target-phase-modulated beta-band power (p = 1.8e–07, Kruskal-Wallis test, H_7_ = 44.44) around the closed-loop target frequency and the phases of maximum amplification and suppression were different (p = 1.2e–07, Watson-Williams test, F_1, 24_ = 55.08), occurring approximately anti-phase (Figures 1E and 1F). Maximally amplifying modulation (2.54 ± 0.93 dB) occurred around the early mid-descending phase (0.38 ± 0.27 p rad), and maximally suppressing modulation (−1.30 ± 0.93 dB) occurred around the early mid-ascending phase (1.35 ± 0.30 p rad). Stimulation at the amplifying and suppressing target phases enhanced and flattened the beta-band spectral peak, respectively (Figure 1G). Absolute target phase also modulated oscillatory power in bands directly above and below the central band (below: p = 2.6e–07, Kruskal-Wallis test, H_7_ = 43.57; above: p = 0.0064, Kruskal-Wallis test, H_7_ = 19.64). The central beta-band power was calculated from the power spectral density (1 Hz bins) using the eleven bins centered on the target frequency bin, with the eleven bins on either side of this central band forming the bands below and above. The out-of-band effect size was larger below than above the central band (Figure 1G), with suppressing stimulation producing amplification at around 26 Hz. Many of the electrodes were located at the striatal GPe border, and such electrodes produced a similar effect size as those fully within (Figure S2). Furthermore, similar modulation could be achieved through stimulation of the subthalamic nucleus (STN) area (Figure S3) only when electrodes were located close to the STN (Figure S4).

### Interplay between stimulus train and oscillation

**Figure 2:**
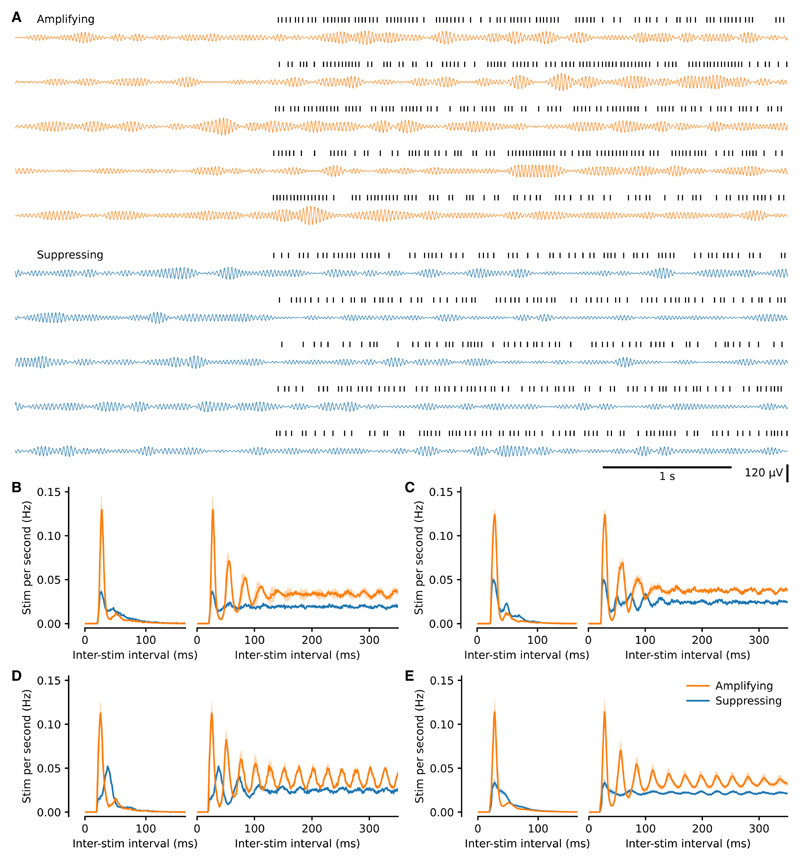
The pattern of stimulation differed between amplifying and suppressing stimulations (A) Example beta-filtered ECoG traces from maximally amplifying and suppressing conditions from the same block. Five consecutive off-to on-epoch transitions in each case. Stimulation times are shown above each trace. Note the difference in stimulation pattern between the two conditions. Beta power is similar during both sets of off-epochs, higher during stimulation targeted to an amplifying phase, and lower during suppressing stimulation yet still waxes and wanes in all conditions. (B) Distribution (mean ± SEM from multiple recording days) of inter-stimulation intervals to the next stimulation (left) and to all subsequent stimulations (right; autocorrelogram) from the same rat as (A). (C and D) Same as (B) for two additional example rats (C and D). (E) Same as (B) for all animals (mean ± SEM, n = 13 rats). Note that the only experimenter-controlled variable change between amplifying and suppressing conditions was selection of a different target phase.

Modulation of beta-band oscillatory power represents a change to the signal that the algorithm uses to calculate phase. Next, we addressed the hypothesis that the feedback loop would produce an interplay between the temporal properties of the oscillation and stimulus train, which would differ between amplification and suppression. The pattern of stimulation was visibly different between these conditions (Figures 1B and 2A), as were the interstimulus interval histograms and autocorrelograms (Figures 2B–2E). We thus investigated if there was a matching difference in the temporal structure of the fluctuations in oscillatory amplitude. Coefficient of variation-type measures when applied to time intervals describe variation in time. However, when directly applied to constant sample rate processes such as oscillatory amplitude, they describe variability in amplitude, not time. Thus, to describe the temporal structure of the oscillations, we developed the temporal variation index (TVI), which is the SD of the derivative of the Hilbert amplitude envelope divided by the envelope mean (see STAR Methods and Figure S5).

**Figure 3:**
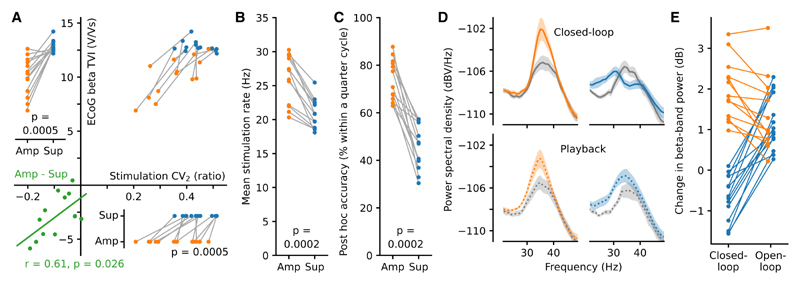
Phase-dependent modulation was produced through brain-machine equilibrium (A) Amplified beta-band oscillations were more stable (lower temporal variation; TVI; n = 13 rats) as were their associated stimulation patterns (lower variation in adjacent inter-stimulus intervals; CV_2_) reflecting the different state of brain-machine equilibrium associated with amplifying, as opposed to suppressing, stimulation (Wilcoxon signed-rank test [WSRT]). The difference between amplifying and suppressing stimulations for both measures was correlated (bottom left, Pearson correlation). (B and C) The different states of brain-machine equilibrium also resulted in differences in mean stimulation rate (B) and post hoc accuracy of stimulation phase (C). P values from WSRT. (D) Power spectra from open-loop playback (bottom) of the closed-loop stimulation patterns (mean ± SEM, n = 27 sessions from 3 rats). (E) Open-loop playback of both suppressing and amplifying closed-loop stimulus trains led to beta amplification (previously amplifying p = 0.0002 and previously suppressing p = 1.2e−04, WSRT), but this was lower in magnitude than closed-loop amplification (p = 0.0007, WSRT, amplifying closed-vs. open-loop).

The amplified oscillation had a lower TVI than the suppressed oscillation, indicating that it was more stable over time (p = 0.0005, Wilcoxon signed-rank test [WSRT], W_13_ = 1; Figure 3A). In line with this, the variation in short timescale dynamics of the stimulation train was lower during amplifying stimulation (CV2; p = 0.0005, WSRT, W_13_ = 1; Figure 3A). Furthermore, the differences in temporal variation of the oscillation and stimulus train between amplification and suppression were positively correlated (p = 0.026, Pearson correlation, r = 0.61; Figure 3A), demonstrating that the stimulus evoked change in the stability of the oscillation was mirrored in the pattern of stimulation itself. Such changes in stimulation pattern likely occurred due to the ability of our system to adapt to the length of each individual cycle and/or not stimulate when the phase estimate was deemed unstable (see STAR Methods). Further evidence of this brain-machine interaction was seen in the difference in stimulation rate (amplifying 25.95 ± 3.34 Hz; suppressing 20.82 ± 2.10 Hz; p = 0.0002, WSRT, W_13_ = 0; Figure 3B) and accuracy (amplifying 73.37% ± 8.48%; suppressing 45.52% ± 9.09% within a quarter cycle of target phase; p = 0.0002, WSRT, W_13_ = 0; Figures 3C and S6). These findings suggested that the phase-dependent modulation of spectral power resulted from the equilibrium that developed between the temporal properties of the beta oscillation and the pattern of the stimulus train.

### Stimulation pattern alone was not sufficient to reproduce the differences in modulation

To test whether the pattern of stimulation alone could result in phase-related power changes, we delivered closed-loop stimulation at amplifying and suppressing phases and then immediately used the recordings to deliver open-loop “playback” of the same stimulation trains (n = 27 recordings, 3 rats). While closed-loop stimulation led to significantly different beta power at amplifying and suppressing phases (p = 1.1e–05, Mann-Whitney rank test, U_13, 14_ = 182), open-loop playback did not (p = 0.75, Mann-Whitney rank test, U_13, 14_ = 98; Figures 3D and 3E). Thus, the temporal relationship between signal and stimulation train was necessary for bidirectional modulation of beta power. Indeed, during open-loop playback, both stimulation trains increased beta-band power (previously amplifying p = 0.0002, WSRT to zero, W_13_ = 0; previously suppressing p = 1.2e–04, WSRT to zero, W_14_ = 0; Figure 3E). It is additionally noteworthy that closed-loop amplification was greater than that produced by open-loop playback (p = 0.0007, WSRT, W_13_ = 2; Figure 3E). Overall, the markedly different spectral modulation produced by closed-loop stimulation highlights the importance of the feedback loop.

### Beta amplification altered the mode of locomotion

**Figure 4:**
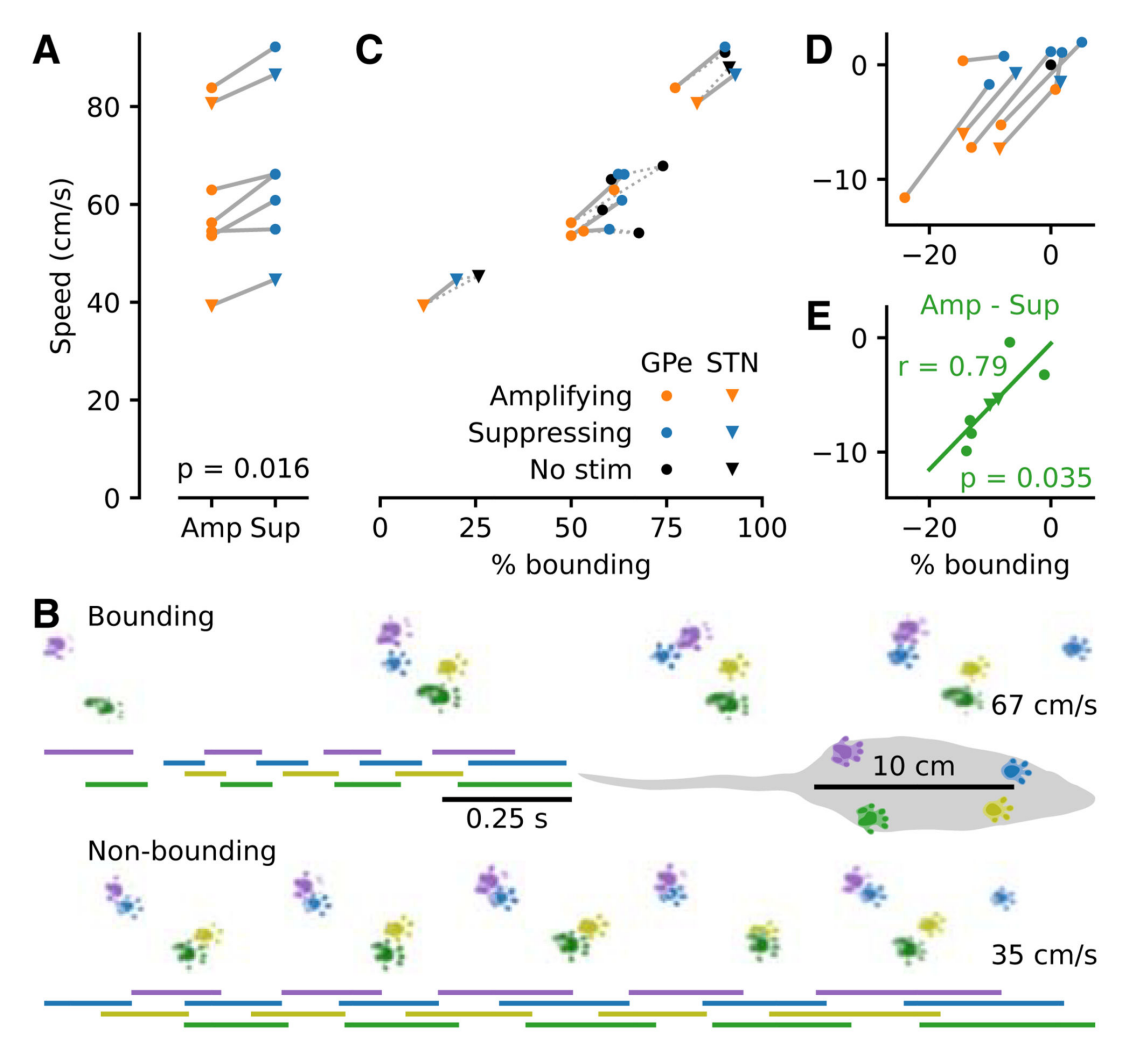
Beta amplification slowed movement speed by altering the mode of locomotion (A) Rats (n = 7) traversed a linear track with reduced mean speed in the presence of amplifying stimulation (WSRT). (B) Same day, same rat example runs (no simulation; one bounding, one non-bounding) visualized from above. Bars depict the time each foot spends in contact with the track. Average speed is noted on the right. The rat covers a similar distance in half the time when bounding. (C) The percentage of bounding runs differed across animals and largely determined mean speed. (D) Same as (C) plotted relative to the no stimulation condition (black dot). (E) The difference in speed between amplifying and suppressing stimulation correlated with the difference in the percentage of bounding runs (Pearson correlation).

Finally, we sought to establish if the neurophysiological differences seen when targeting stimulation to an amplifying, as opposed to a suppressing, phase were functionally relevant. Beta oscillations are associated with slowing or holding of ongoing movement (Engel and Fries, 2010). We hypothesized that the different modes of beta modulation generated by our system would lead to accompanying changes in behavior. To test this, speed and gait parameters were evaluated while a subset of GPe- and STN-stimulated animals (n = 7) traversed a linear track. Recordings were between 5 and 20 min in duration and typically consisted of eight valid runs with multiple groups of recordings per animal collected across different days. Stimulation was continuously enabled throughout. Amplification, as opposed to suppression, of cortical beta power significantly reduced the speed at which animals moved across the linear track (p = 0.016, WSRT,W_7_ = 0; Figures 4A and S7). Rodent locomotion can be categorized into distinct gaits that can be broadly divided into slower alternating limb patterns (e.g., walking and trotting) and faster “bounding” patterns, where the forelimbs and hindlimbs are moved synchronously and landed together (Bellardita and Kiehn, 2015). We classified a run as being bounding if it exclusively contained a bounding gait for all strides. The remaining valid runs were classified as non-bounding. This included runs with at least one alternating stride in addition to runs with exclusively alternating gait. We then examined the expression of these locomotor patterns across stimulation protocols. This analysis revealed that the speed change was caused by a reduction in the ratio of faster bounding-based movement to slower alternating-based movement, irrespective of the animal’s affinity for bounding without stimulation (Figures 4B–4E; speed difference correlated with difference in percentage of bounding p = 0.035, Pearson correlation, r = 0.79). Beta amplification therefore elicited a change in the mode of locomotion, leading to slower movement.

## Discussion

Closed-loop stimulation has enormous potential to improve the treatment of brain disorders (Cagnan et al., 2019a). The majority of closed-loop applications thus far have attempted to detect the occurrence of a biomarker of disease and disrupt it, with the aim of ameliorating the associated symptoms. Here, we describe a fundamentally different approach. We demonstrate that phase-dependent interaction with an oscillatory biomarker on fast timescales (milliseconds) can establish a flexible brain-machine equilibrium, resulting in a sustained amplification or suppression of the target activity. By comparing closed- and open-loop stimulation protocols, we demonstrate unambiguously that this interaction was necessary for bidirectional modulation, which could not be induced using the stimulation patterns alone. Crucially, interactive modulation of ongoing beta activity resulted in a change in behavior, altering the speed of movement by biasing the locomotor pattern. Together, these findings demonstrate that maintaining a continuous interaction between an external system and brain activity could be used to drive brain and behavior toward a desired state.

Cortical circuits entrain widespread populations of neurons across subcortical nuclei, which project directly or indirectly back to the same cortical areas. Such connectivity has been proposed to underlie the generation of beta-frequency synchronization across cortico-basal ganglia circuits (Brazhnik et al., 2016; Cagnan et al., 2019b; Mirzaei et al., 2017). Delivering phase-locked stimulation that exploited the temporal relationship between cortical and basal ganglia populations was sufficient to fundamentally change the power and stability of the cortical oscillation. These changes were mirrored by the pattern of the stimulus train, thus an equilibrium emerged, determined by the fixed parameters of the system and the properties of the brain network. Furthermore, altering the phase of stimulus delivery was sufficient to adjust the point of equilibrium. Open-loop playback of the suppressing stimulus train led to amplification of the cortical beta oscillation, presumably due to the oscillatory pattern of the stimulus. This reversal in effect clearly illustrates the temporal dependence of signal and stimulus in preventing the oscillation from emerging rather than the primary statistics of the stimulation train. Similarly, stimulation at the amplifying phase reinforced the stability of the ongoing oscillation more than could be achieved by the stimulus pattern alone. The system, therefore, worked like an external node, simultaneously adapting to and influencing the state of the network.

An outstanding question is how this brain-machine interaction manifests at the level of single units in the cortex and basal ganglia. Several factors are likely to contribute to the relationship between underlying spiking activity and suppression or amplification. For example, specific phases of the ECoG could have fixed relations to the absolute refractory period of a large number of synchronized neurons, perhaps of a particular cell type. This could make the stimulus more or less likely to reinforce oscillatory spiking both locally and downstream. The phase could also represent when a stimulus is more likely to reinforce or disrupt spike-timing synchrony across the local population or in relation to the rest of the network. In all cases, which are not mutually exclusive, the stability generated by the closed-loop interaction could act to maintain a specific temporal relationship between spiking and the ECoG. Unit recordings would also enable further investigation of off-band effects, whereby suppressing stimulation led to an increase in the power of neighboring, lower frequencies. Such effects were also sometimes visible in the pattern of the stimulation pulses, whereby suppression slowed the frequency of stimulation with respect to the target frequency (e.g., Figure 2D). Previous work in 6-OHDA-lesioned rats has shown that basal ganglia spiking is locked to beta oscillations at the target frequency (Brazhnik et al., 2014). A key question is whether this spiking activity also moves to lock to the shifted oscillatory peak frequency or only disengages from the target band. Recordings of spiking across the network will ultimately be required to address these questions.

The change in locomotion between amplifying and suppressing stimulation—where the only difference between the two conditions was the phase to which the stimulation was targeted—demonstrates that the different states of equilibrium resulted in functionally relevant changes in brain state. Amplification influenced the locomotive preference toward walking over bounding, yet the inter-animal variation in bounding and walking still occupied a similar range to the no-stimulation condition. This suggests that the stimulation did not completely disrupt normal function but instead biased the natural operation of the network, resulting in a shift in behavioral tendencies. Beta oscillations in cortico-basal ganglia networks are associated with a holding of the current motor state (Brittain and Brown, 2014; Engel and Fries, 2010) rather than with a specific type of movement or behavior. Indeed, the patterned limb movements needed for walking or bounding are mostly coordinated in the brainstem and spinal cord (Ferreira-Pinto et al., 2018). As the rats turned in the reward arena prior to entering the track, they could only move using a walking-like locomotor pattern. Increased beta activity in cortico-basal ganglia networks potentially served to maintain the walking motor program, reducing instances of clean transition into bounding across the track. This ability to bias ongoing behavior may be particularly advantageous for proposed translational applications of phase-dependent stimulation, where the goal is to shepherd dysfunctional networks away from pathological cognition and behavior (Takeuchi and Berényi, 2020; Widge and Miller, 2019).

Our work supports the idea that integrating an external system within the intrinsic dynamics of a pathophysiological neural circuit can provide a “network prosthesis,” whereby the closed-loop system compensates for the myriad of maladaptive changes that prevent the network from functioning within its normal range. Moreover, we demonstrate that it is possible to achieve this by interacting with the brain at millisecond timescales and, in turn, that this timescale is functionally important. We have provided tools to implement these methods in preclinical experiments and in medical devices. The approach is already clinically tractable by combining the fast real-time algorithm used here with a next-generation DBS device (Gilron et al., 2021; Opri et al., 2020; Toth et al., 2020). Our demonstration that network oscillations can be manipulated bidirectionally, and within their normal functional range, provides a technical and conceptual approach to define the role of these activities in the function and dysfunction of memory, sleep, and other fundamental brain operations.

## Limitations of the study

We targeted two basal ganglia structures known to be highly coupled to cortical beta oscillations (Cagnan et al., 2019b; Crompe et al., 2020; Mallet et al., 2008). While we were able to show that closed-loop stimulation targeted to the STN can lead to similar physiological and behavioral effects to those of the GPe, more experiments will be needed to fully characterize the amplifying and suppressing phases of STN stimulation to allow comparison of the two targets. A more general limitation is that we cannot be sure which neuronal elements around the stimulation electrode were most important in modulating cortical oscillations. It is important to acknowledge that we do not claim that it was necessary for the stimulating electrode to be within the target structure to see electrophysiological and/or behavioral effects of closed-loop stimulation. As with our own complementary work on phase-dependent effects of stimulation in people with PD (Holt et al., 2019), we expect effects on neurons in the target areas, surrounding areas, and fibers of passage to all have the potential to disrupt or reinforce cortico-basal ganglia oscillations. Dissection of exactly which of these components is necessary or sufficient to modulate cortical beta power would require our methods to be combined with optogenetic stimulation. As in clinical DBS, however, the less-specific effects of electrical stimulation may enable multiple routes of modulation, which is not necessarily a disadvantage when trying to manipulate a large network.

The oscillations that we measured and manipulated here had a center frequency of around 35 Hz. In most other systems and contexts, this frequency would be considered low-gamma range (Buzsáki and Draguhn, 2004). However, frequencies up to 35 Hz are often considered part of the extended beta range in studies of patients with PD (Baaske et al., 2020; Iskhakova et al., 2021; Kühn et al., 2006; Neumann et al., 2016; Sharott et al., 2018), and this higher range has been repeatedly shown to be the dominant frequency in the awake, 6-OHDA-hemi-lesioned rat (Avila et al., 2010; Brazhnik et al., 2014; Sharott et al., 2005). It should be acknowledged, however, that patients with PD generally have considerably lower dominant frequencies and that these may be the most clinically relevant (Darcy et al., 2022; Neumann et al., 2016). Importantly, the main findings of this study relate to the interactive properties of our system when coupled to a pronounced neural oscillation and would likely translate to the lower beta range or other bands.

## Star★Methods

### Key Resources Table

**Table:** 

REAGENT or RESOURCE	SOURCE	IDENTIFIER
Antibodies		
Rabbit anti-TH primary	Millipore	Cat# AB152; RRID:AB_390204
Guinea pig anti-parvalbumin primary	Synaptic Systems	Cat# 195-004; RRID:AB_2156476
Rabbit anti-FoxP2 primary	Sigma	Cat# HPA000382; RRID:AB_1078908
Donkey anti-rabbit Alexa Fluor 488 secondary	Thermo Fisher Scientific	Cat# A-21206; RRID:AB_2535792
Donkey anti-guinea pig Cy3 secondary	Jackson ImmunoResearch	Cat# AB_2340460; RRID: AB_2340460
Chemicals, peptides, and recombinant proteins		
6-hydroxydopamine (6-OHDA)	Sigma; Bio-techne	Sigma: H4381-100MG; Bio-techne: 2547/50
Deposited data		
Electrocorticogram with closed-loop stimulation recordings and associated data	This paper	https://data.mrc.ox.ac.uk/ecog-closed-loop; https://doi.org/10.5287/bodleian:9omadD7Pp
Experimental models: Organisms/strains		
Lister Hooded rats	Charles River	strain code 603
Software and algorithms		
OscillTrack	This paper	https://colinmcn.github.io/OscillTrack/; https://doi.org/10.5287/bodleian:qa9ngXrzr
SciPy	scipy.org; conda-forge.org	Version 1.8.1
Pycircstat	pypi.org	Version 0.0.2
Matplotlib	matplotlib.org; conda-forge.org	Version 3.5.2
CatWalk XT	Noldus, Netherlands	Version 10.6.608
Inkscape	inkscape.org	Version 1.1.2

## Resource Availability

### Lead contact

Further information and requests for resources should be directed to and will be fulfilled by the lead contact, Andrew Sharott (andrew.sharott@bndu.ox.ac.uk).

### Materials availability

This study did not generate new unique reagents.

## Experimental Model and Subject Details

Male Lister Hooded rats (starting weight 350–450 g; estimated starting age 3–4 months; experiment duration 2–4 months; Charles River, strain code 603) were housed with free access to food and water in a dedicated housing room with a 12/12-h light/dark cycle. Closed-loop stimulation, data acquisition and behavioural testing took place in a separate room during dedicated recording sessions. Experiments involving animals were conducted in accordance with the UK Animals (Scientific Procedures) Act 1986 under personal and project licenses issued by the Home Office following ethical review.

## Method Details

### Surgical procedures

Rats underwent two separate recovery stereotaxic surgical procedures performed under deep anaesthesia using isoflurane (4% induction, 2–0.5% maintenance) and oxygen (2 L/min). Local anaesthetic (Marcaine, 2 mg/kg, 2.5 mg/mL) and non-steroidal antiinflammatories (Metacam, 1 mg/kg, 5 mg/mL) were administered subcutaneously at the beginning of all surgeries, while opioid analgesia (Vetergesic, 0.3 mg/mL, 0.03 mg/kg) was also provided for three consecutive post-operative days.

The first surgical procedure produced a unilateral lesion of dopaminergic neurons of the substantia nigra pars compacta by intracranial injection of the neurotoxin 6-hydroxydopamine (6-OHDA) at their cell bodies through a glass pipette located 4.9 mm posterior and 2.3 mm lateral from bregma at a depth of 7.8 mm from the brain surface. 6-OHDA (Sigma: H4381-100MG; Bio-techne: 2547/50) was dissolved immediately before use in phosphate buffer solution containing 0.02% w/v ascorbate to a final concentration of 6 mg/mL. Between 0.10 and 0.15 μL of 6-OHDA solution was injected at a rate of 0.01 μL/min through the pipette, which was left in place a further 5 min before being withdrawn. Front-foot use asymmetry pre- and post-lesion while rearing against the wall of a transparent acrylic cylinder was used to indicate lesion severity and select candidates for electrode implant.

Following full recovery and not less than 13 days later, selected animals underwent a second surgical procedure to implant two pairs of stainless steel stimulation electrodes (California Fine Wire, stainless steel, bifilar, heavy formvar insulation, 127 μm strand diameter). Electrodes were secured with bone cement and six M1.4X3 mm stainless steel screws. For stimulation of the globus pallidus (GPe), electrodes were implanted 1 mm posterior and 3.1 mm lateral from bregma at a depth of 6 mm from the brain surface. For stimulation of the subthalamic nucleus (STN) electrodes were implanted 3.8 mm posterior and 2.5 mm lateral from bregma at a depth of 7.2 mm from the brain surface. ECoG was measured from the most frontal screw located above motor cortex at approximately 4.6 mm anterior and 1.6 mm lateral from bregma referenced to two screws above cerebellum. Neurotoxin injections, electrical stimulation and ECoG recording were all performed on the right hemisphere.

### Immunohistochemistry and electrode location

Upon completion, rats were deeply anesthetized with isoflurane (4%) and pentobarbital (3 mL, Pentoject, 200 mg/mL) and transcardially perfused with phosphate-buffered saline (PBS) followed by fixative (paraformaldehyde dissolved in PBS, 4%, wt/vol). With stimulation wires still in place, heads were placed in fixative for a further 12 hours before brains were extracted and sectioned. Lesions were verified using antibodies to tyrosine hydroxylase (rabbit anti-TH primary; diluted 1:1000; Millipore Cat# AB152, RRID:AB_390204; donkey anti-rabbit Alexa Fluor 488 secondary; diluted 1:1000; Thermo Fisher Scientific Cat# A-21206, RRID:AB_2535792) to visualise the unilateral loss of cell bodies in the SNc and loss of dopaminergic innervation in the dorsal striatum. Sections containing electrode tracks were stained for either parvalbumin (guinea pig anti-parvalbumin primary; diluted 1:1000; Synaptic Systems Cat# 195-004, RRID:AB_2156476; donkey anti-guinea pig Cy3 secondary; diluted 1:500; Jackson ImmunoResearch Cat# AB_2340460, RRID: AB_2340460) or FoxP2 (rabbit anti-FoxP2 primary; diluted 1:500; Sigma Cat# HPA000382, RRID:AB_1078908; donkey anti-rabbit Alexa Fluor 488 secondary; diluted 1:1000; Thermo Fisher Scientific Cat# A-21206, RRID:AB_2535792) to determine electrode location in relation to the GPe and STN respectively. Stimulation electrode tracks were visualised with epifluorescence microscopy. Electrode tips classified as at the GPe striatal border were a maximum of 600 μm from the border. The electrode located above the STN was 250 μm from the dorsal border and the two electrodes located below the STN were both 450 μm from ventral border.

### Open field eight phase protocol

Rats freely explored a 90 by 50 cm dimly lit open field surrounded on three sides, above and below with electrical shielding with a black curtain for access on the remaining side. Three to six recording blocks were performed per rat. Recording blocks consisted of eight pairs of recordings with a different real-time target phase applied in each pair (Figure S1A). Target phase order was randomised across blocks. Stimulation was enabled in the first recording of each pair (stimulation recordings) and disabled in the second (baseline recordings). Stimulation recordings were approximately 4.5 min in duration and baseline recordings were approximately 2 min in duration. The real-time system generated triggers during 20 second epochs (on-epochs) separated by 5 second trigger free epochs (off-epochs) and was in operation across all recordings. Recordings were made both when triggers were used to drive stimulation and with stimulation disabled. Generating real-time triggers in the absence of stimulation during baseline recordings allowed comparison of the real-time system performance of phase tracking with and without stimulation influencing the neural activity. These recordings also provided the opportunity to assess baseline physiology in the absence of stimulation. Embedding short epochs lacking stimulation within recordings with stimulation provided an internal comparison point to assess the effect of stimulation without the need to correct for changes occurring over longer time frames such as arousal, brain state, general movement levels and external electrical noise. The centre frequency (f_c_) for real-time phase tracking for each block was chosen at the start of the block based on power spectra generated from previous recordings from the same rat and was in the range 35 to 41 Hz.

### Electrocorticogram and electrical stimulation

Electrocorticogram (ECoG) signals measured from M1.4 stainless steel screws referenced to two cerebellar screws were amplified and digitised using a RHD2000 family amplifying and digitising headstage connected to a RHD USB Interface Board both from Intan Technologies (intantech.com). The USB Interface Board was recently discontinued and an RHD recording controller is the recommended replacement. Electrical stimulation was driven using a fully isolated current source (battery powered with an optically coupled trigger, A365RC, World Precision Instruments) with the source and sink terminals connected to the adjacent strands of the pair of stainless steel wires forming the stimulation electrode. Stimulation events were biphasic consisting of two consecutive pulses of opposite polarity each 50, 60 or 70 μA in amplitude and 95 μs in duration separated by 10 μs. To achieve closed-loop stimulation, a custom designed digital circuit was used to access the digital data stream from the headstage, track phase within a band of interest in real-time and generate digital pulses to activate the optically coupled trigger of the stimulation current source. The custom designed digital circuit was implemented using extra available digital circuitry within the field programmable gate array (FPGA) located on the RHD USB Interface Board.

### Real-time phase tracking and closed-loop trigger algorithm (OscillTrack)

To perform real-time oscillatory tracking, we developed the OscillTrack algorithm, which we use for the first time here. In addition to the described FPGA implementation, we also provide an alternative implementation in the form of microcontroller code (https://colinmcn.github.io/OscillTrack/). The approach seeks to match the measured signal to a fixed model (a pair of constant frequency sine and cosine waves) to build a signal estimate. In this sense, it can be thought of as a partial implementation of a Kalman filter with modifications for application-specific functionality and computational efficiency. By design, the measurement and process noise are not explicitly modelled and the error update coefficient (*γ*, see below) is set constant by the user, versus the explicit calculation of a Kalman gain term. Intuitively, the update coefficient sets the width of the passband allowing the user to select a value to suit their application.

ECoG recordings were performed with a sample rate of 20 kHz per channel to capture the full detail of stimulation artefacts aiding their removal in post hoc analysis. However, for phase tracking in real-time, the data was downsampled by producing a single sample from the sum of 8 to 12 (*N_dn_*) successive samples, since tracking beta-band phase did not require such a high number of samples per cycle. Thus, the phase tracking component operated at sample rates in the range 1.67 to 2.5 kHz (varying the sample rate was one of the ways to achieve different target oscillation centre frequencies).

To track phase in real-time, an iterative algorithm was applied where in each step (*n*) we estimated the phase (*φ_n_*) based on the input signal (*s_n_*) as follows. A pair of continuously oscillating constant frequency sine and cosine reference waves (sin *θ_n_* and cos *θ_n_*) were generated from a precomputed quarter cycle lookup table containing *N* values such that:

$$\theta_{n}=\frac{n\pi }{2N}$$

A band limited complex estimate of the signal (*r_n_*) was calculated using a pair of weighting coefficients (*α_n_* and *β_n_*) that jointly determined the phase and amplitude of the estimated signal with respect to the reference waves as follows:

$$r_{n}=\left(\alpha_{n}\sin\theta_{n}+\beta_{n}\cos\;\theta_{n}\right)+i\left(\beta_{n}\;\sin\;\theta_{n}-\alpha_{n}\;\cos\;\theta_{n}\right)$$

On each iteration the weighting coefficients were updated to minimize the error (Δ_*n*_) between the input signal (*s_n_*) and the real part of the estimate (Re(*r_n_*)).

$$\Delta_{n}=s_{n}-\text{Re}\left(r_{n}\right)$$

Specifically, the weighting coefficients were updated for the next iteration such that:

$$\begin{array}{c} \alpha_{n+1}=\alpha_{n}+\gamma \Delta_{n}\;\sin\;\theta_{n}\\ \beta_{n+1}=\beta_{n}+\gamma \Delta_{n}\;\cos\;\theta_{n} \end{array}$$

The convergence between the estimate and the signal was thus limited by a constant coefficient (*γ*) on the error update terms, which in these experiments was set to 2^−4^. Choice of a negative power of two reduced the computation required from a multiplication to a bitwise right shift. The real-time phase estimate (*φ_n_*) was generated from *r_n_* using an 11 stage CORDIC (Volder, 1959) comprising entirely of combinatorial logic.

$$\varphi_{n}=\text{angle}\left(r_{n}\right)$$

The centre frequency (*f_c_*) of the band of interest was set by the frequency of the reference waves (sin *θ_n_* and cos *θ_n_*). As described, this depended on the original sample rate (20 kHz), the amount of downsampling (*N_dn_*), and the number of values in the lookup table containing a quarter of a sine wave (*N*).

$$f_{c}=\frac{20000}{4N\;N_{dn}}\;\text{where}\;N\in \left\{11,...,15\right\}\;\text{and}\;N_{dn}\in \left\{8,...,12\right\}$$

The width of the band of interest was set by the error update coefficient (*γ*), which damped the rate of convergence between the estimate and the signal. The only state information retained between iterations was thus the weighting coefficients (*α*_*n* +1_ and *β*_*n* + 1_) and the reference wave lookup index which was incremented by one each iteration and reset on completion of a reference wave cycle. The operations described above were implemented as dedicated combinatorial logic.

A real-time trigger was generated when the phase estimate passed into the target phase range unless it had been less than 0.8 of a target frequency (*f_c_*) period since the most recent passing into the target phase range. This was important to reduce stimulation at times of poor phase estimates caused by low power in the target frequency range. Additionally, a phase shift corresponding to half the stimulation width (100 μs) was subtracted from the target phase causing the stimulation to be triggered slightly early allowing the middle of the stimulation to occur at the desired target phase. To reduce the effect of electrical stimulation artefacts on the phase estimate, a digital hold circuit operating on the 20 kHz data stream held the sample preceding the stimulation trigger on the input of the downsampling circuit for 600 μs from the start of the trigger. An offset removal digital filter was also implemented on the input to the phase tracking algorithm to remove residual offset remaining from the analogue front-end electronics; for each sample step (*n*) the offset removed signal (*s_n_*) was generated from the downsampled original signal (*q_n_*) whereby:

$$\begin{array}{c} s_{n}=q_{n}-x_{n}\\ x_{n+1}=x_{n}+2^{-6}s_{n} \end{array}$$

### Data analysis

First, stimulation artefacts were removed from the 20 kHz recordings by interpolation between the sample immediately preceding the stimulation and the sample 1.7 ms later. The resulting signals were downsampled to 1 kHz in two steps using finite impulse response anti-aliasing filters (designed using scipy.signal.remez(), combined pass band ripple less than 0.001 dB below 420 Hz, stop band attenuation greater than 90 dB above 498 Hz). All spectra and phase calculations were performed on the artefact-removed and downsampled signal. Post hoc trigger phase in the beta-band was calculated by filtering (scipy.signal.firwin(), numtaps = 513) the signal between ±5 Hz of the centre frequency (f_c_) chosen for real-time phase tracking. The trigger phase was then calculated as the polar angle of the Hilbert transform analytic signal at the midpoint of the biphasic trigger. Power spectral density (PSD) calculations were performed using Welch’s method (Welch, 1967) with a resolution of 1 Hz spectral bins. Changes in beta-band power were calculated from the respective PSDs (mean of f_c_ bin ± 5 bins inclusive, i.e. mean of 11 bins total) in dB. For capturing the variation in stimulation dynamics, coefficient of variation 2 (CV_2_) was used (Holt et al., 1996). It was calculated as the mean value across the stimulation train of two times the absolute value of the difference over the sum of adjacent stimulation intervals. It has a value of one for a Poisson process and zero for regularly spaced (fixed frequency) stimulation.

### Temporal variation index

In order to quantify the interplay between the stimulus train and the beta oscillation, we required a measure of the oscillatory variability in amplitude with respect to time. Coefficient of variation-type measures when applied to time intervals, capture the variability in time for point processes such as neuronal spiking or stimulation trains. However, for constant sample rate processes such as oscillatory amplitude they cannot be directly applied to the time axis and give a measure of the variability in amplitude, not time. Consider two identical signals with one difference; one is progressing at twice the rate of the other. Their variance and coefficient of variation (CV) in amplitude would be identical. However, the variance in their rate of change would differ and this is the property we sought to measure. The more slowly progressing signal could be described as more stable as its amplitude changes more slowly. We thus developed the temporal variation index (TVI; Figure S5) where the differential of the signal is taken before variance is measured. Differentiation of such a signal results in values that are normally distributed being centred on zero and hence can be fully described by their standard deviation (see Figure S5 bottom row). A second requirement was that the measure should be independent of mean amplitude as we wished to compare the temporal dynamics between the amplified and suppressed signals. This was achieved by normalisation by the mean. Without normalisation, signals with a higher mean would have a higher TVI. This normalisation is equivalent to that to produce CV. Thus, we defined TVI as the standard deviation of the derivative of the Hilbert amplitude envelope divided by the envelope mean. A more stable signal will have a lower TVI and a more variable signal will have a higher TVI.

### Linear track gait analysis

Gait analysis during closed-loop stimulation was performed using a customised version of the CatWalk XT (Noldus, Netherlands). The CatWalk system consists of a glass walkway raised above a camera capturing at 100 fps. The glass plate is illuminated by green light that reflects within the glass at points being touched, allowing for semi-automated detection of footprints as rats traverse the walkway. The CatWalk was modified to include arenas extending from the ends of the track each containing a reward port. Aluminium sheeting was attached along the walls of the track to provide electrical shielding and increase the wall height. The red strip light above the track was replaced with red ambient lighting allowing the addition of a zip line to accommodate the recording and stimulation tether.

Sugar pellets were delivered by the experimenter through dedicated tubing to the reward delivery ports at alternating ends of the track. During training, tethered rats were allowed to freely explore the glass walkway and reward areas for approximately four fifteen-minute sessions until they consistently crossed the walkway to collect reward. Amplifying and suppressing stimulation phases and stimulation target (GPe or STN) for each rat were selected based on spectra generated from open field eight phase recordings. During data acquisition, no stimulation, amplifying-phase stimulation and suppressing-phase stimulation were applied in separate recordings in a randomised order. Recordings were between five and twenty minutes in duration and typically consisted of eight valid runs with multiple groups of recordings per animal collected across different days. Gait analysis was performed on rats in which successful amplification and suppression was achieved during linear track recordings. Damaged implants prevented collection of linear track data from two of the 13 rats comprising the open field data. Post hoc analysis of linear track ECoG data revealed that amplification and suppression of beta-band power did not occur in a further four rendering the data unsuitable for the comparison of amplifying and suppressing stimulation (two had no modulation and the incorrect target phases were selected during data collection for another two). Of the remaining seven included in gait analysis, five received stimulation of the GPe and two received stimulation of the STN.

Paw prints were detected and descriptive properties generated using CatWalk XT software (Noldus, Netherlands, version 10.6.608) and the resulting output further analysed using SciPy (scipy.org). Only runs less than four seconds in duration having at least eight valid footprints and an average speed of greater than 10 cm/s were included in the analysis. To identify faulty recordings, the total spectral power below 200 Hz was calculated for each recording and those in the upper tail of the resulting distribution were excluded (20 of 420). The average number of valid runs per condition per animal was 100.7 with a minimum of 69. Average speed for each run was calculated as the average distance travelled divided by time taken for each step cycle i.e. from start of contact to start of next contact for each cycle of each foot (CatWalk XT). Runs were classified as bounding if each forelimb contact was followed or preceded by the other across the entire valid run with the same being true of hindlimbs e.g. front-front-hind-hind-front-front etc., never front-hind-front (CatWalk XT 100 % cruciate or rotate). The percentage bounding was the percent of total valid runs for that condition that were classified as bounding.

## Quantification and Statistical Analysis

Post hoc analyses of ECoG spectral properties, stimulation phase and behavioural data including statistical tests were performed using SciPy (scipy.org; version 1.8.1). The Watson-Williams test was performed using pycircstat (pypi.org/project/pycircstat version 0.0.2). Values in the text are reported as mean ± standard deviation and error bars in plots show mean ± standard error of the mean unless otherwise stated. Nonparametric tests were used and each test is specified in the results text along with the p-value, associated test statistic, degrees of freedom and n.

## Supplementary Material

Supplementary Material

## Data Availability

Electrocorticogram recordings are available from the MRC Brain Network Dynamics Unit Data Sharing Platform/Oxford University Research Archive (https://data.mrc.ox.ac.uk/ecog-closed-loop; https://doi.org/10.5287/bodleian:9omadD7Pp). Additional data reported in this paper will be shared by the lead contact upon request. All original code is available from (https://colinmcn.github.io/OscillTrack/; https://doi.org/10.5287/bodleian:qa9ngXrzr) including Hardware Description Language for the OscillTrack algorithm along with alternative Microcontroller C code. Any additional information required to reanalyze the data reported in this paper is available from the lead contact upon request.

## Highlights

- Closed-loop bidirectional modulation of beta power by phase-specific stimulation
- Equilibrium develops between the dynamics of the oscillation and the system output
- Target phase dictates equilibrium state and level of amplification or suppression
- Beta amplification slows ongoing movement by biasing the animal’s locomotor pattern
