# Supplementary material for: Stable, interactive modulation of neuronal oscillations produced through brain-machine equilibrium
Source: Cell Rep. Author manuscript; Available in PMC 2023 Jan 21. (PMC7614081; doi:10.1016/j.celrep.2022.111616)
Supplement: Supplementary Material [file EMS159400-supplement-Supplementary_Material.pdf]

Cell Reports, Volume *41*

Supplemental information

Stable, interactive modulation of neuronal oscillations produced through brain-machine equilibrium

Colin G. McNamara, Max Rothwell, and Andrew Sharott

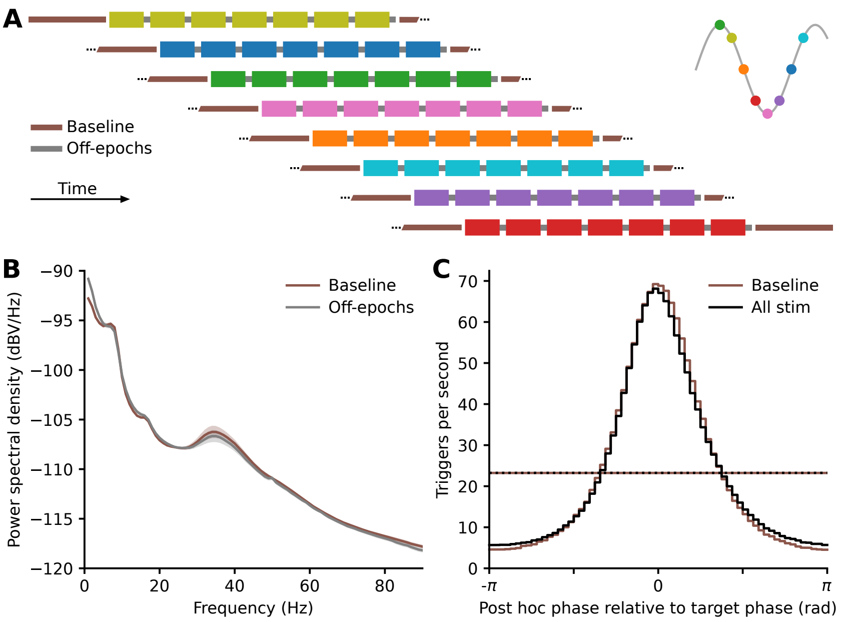

Figure S1. Phase-dependent stimulation targeting parkinsonian beta oscillations.
Related to STAR Methods and Figure 1.
(A) Schematic of a recording block. Closed-loop stimulation occurred during 20 second epochs (on-epochs; colours) separated by 5 second trigger free epochs (off-epochs, grey). Baseline periods (brown) without stimulation were also recorded between each stimulation recording. Target phase (colours, see insert) order was randomised across blocks.
(B) Power spectral density plot (mean ± SEM, n = 13 rats) showing a clear peak in beta-band power in the absence of stimulation recorded from frontal cortical ECoG screws located ipsilateral to the 6-OHDA lesion. Elevated beta-band power in off-epochs (grey) was similar to that seen in baseline recordings (brown).
(C) Trigger accuracy histogram of ECoG phase calculated post hoc plotted relative to target phase. The accuracy and mean trigger rate (dotted lines) of the real-time generated triggers, without stimulation enabled (brown) and with stimulation delivered to the globus pallidus (black) were almost identical, demonstrating that the system was able to maintain full accuracy in the presence of electrical artefacts and physiological perturbation produced by stimulating. Note, post hoc phase analysis uses future information to calculate phase (primarily due to the band pass filter) and thus by definition represents a different measure to real-time phase regardless of the real-time algorithm used.

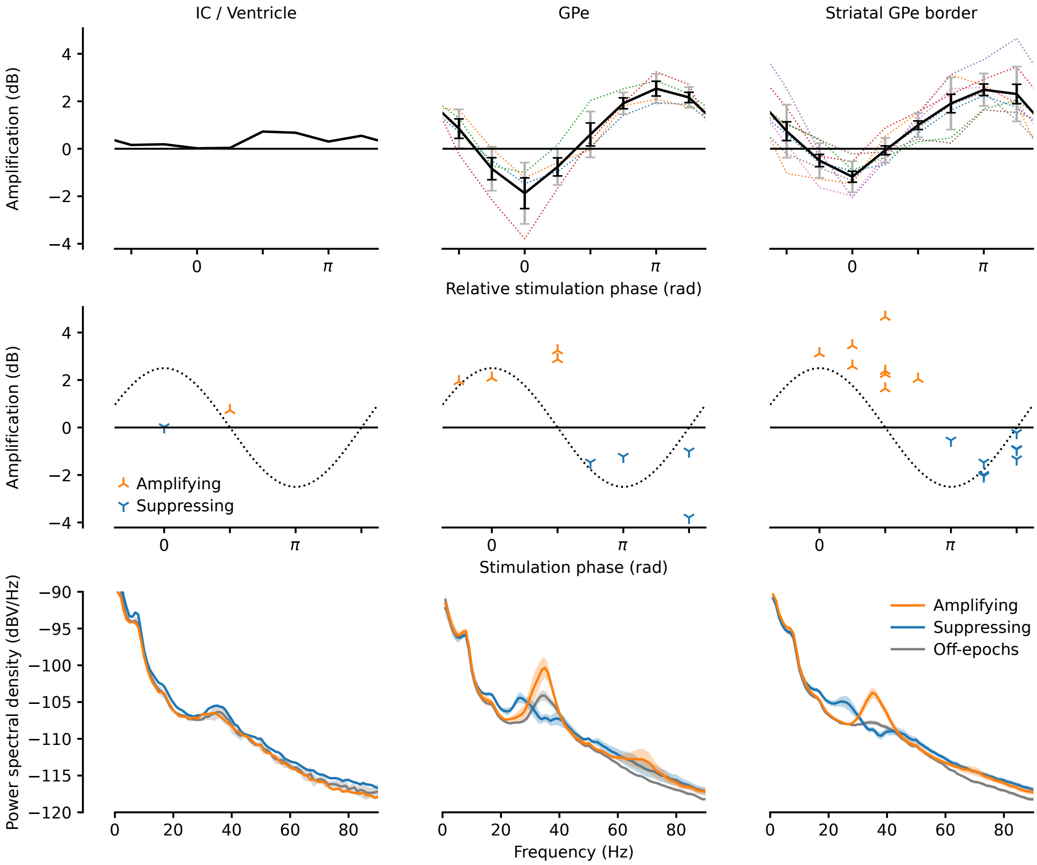

Figure S2. Modulation grouped by electrode location for stimulation targeted to the GPe.
Related to Figure 1.
Modulation measures with stimulation electrode located at the internal capsule lateral ventricle border (left column), within the GPe (middle column) and at the striatal GPe border (right column). Top row, change in beta-band power (on- compared to off-epochs) due to stimulation for 8 target phases aligned by the most suppressing target phase for each rat. Dotted lines show the values for each rat with mean ± SEM in black and standard deviation in grey. Middle row, absolute phase versus change in beta-band power of the most suppressing and most amplifying target phases for each rat. The dotted line represents the beta-cycle. Bottom row, power spectral density plots (mean ± SEM).

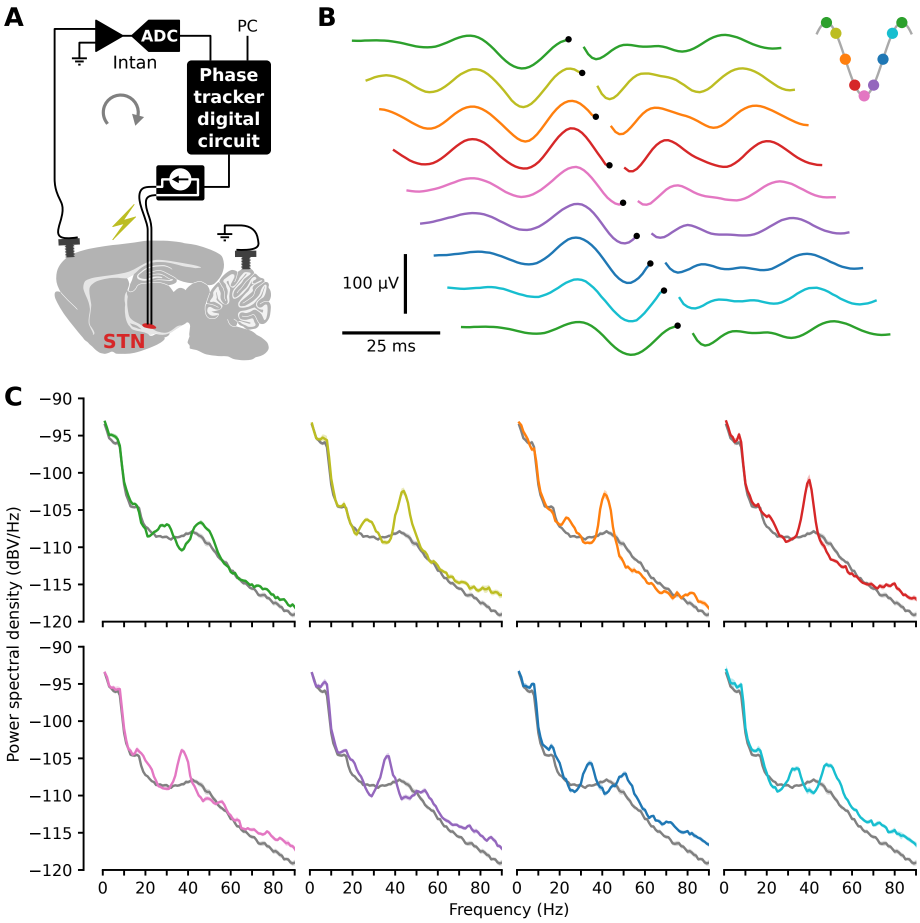

Figure S3. Phase-dependent modulation through stimulation of the subthalamic nucleus.
Related to Figure 1.
(A) A digital circuit triggered delivery of electrical pulses to bipolar electrodes just above the subthalamic nucleus at a predetermined ECoG beta-band phase.
(B) Stimulation triggered averages from an example block of ECoG recordings. Stimulation was targeted to eight equally spaced phases across separate recordings. To aid visualisation, trigger times (black dots) are staggered across traces and the first trace is repeated.
(C) Power spectra from stimulation on-epochs for each target phase (colours as in **B**) from the same example recordings and for all off-epochs embedded in those same recordings (grey).

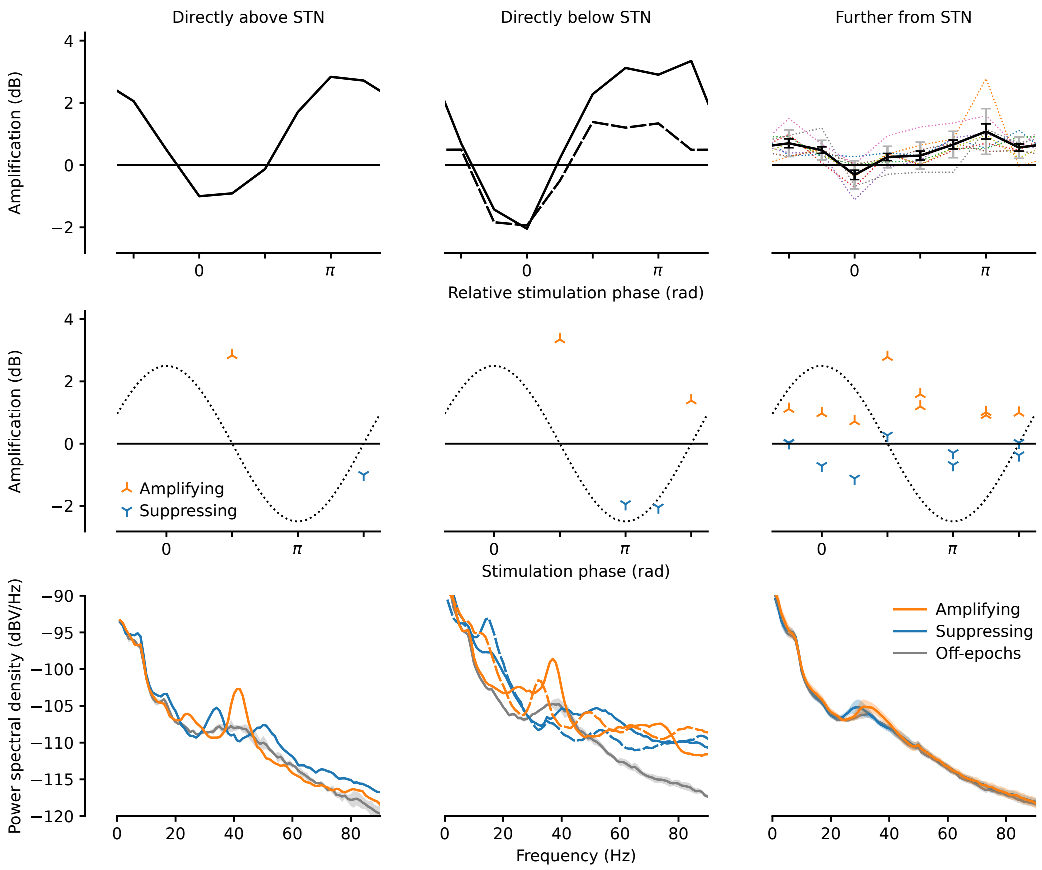

Figure S4. Modulation grouped by electrode location for stimulation targeted to the STN area.
Related to Figure 1.
Modulation measures with stimulation electrode located 250 µm above the STN (left column), 450 µm below the STN (middle column) and further from the STN (right column). Top row, change in beta-band power (on- compared to off-epochs) due to stimulation for 8 target phases aligned by the most suppressing target phase for each rat. One trace per rat (mean ± SEM in black and standard deviation in grey on right plot only). Middle row, absolute phase versus change in beta-band power of the most suppressing and most amplifying target phases for each rat. The dotted line represents the beta-cycle. Bottom row, power spectral density plots (mean ± SEM where error is shown).

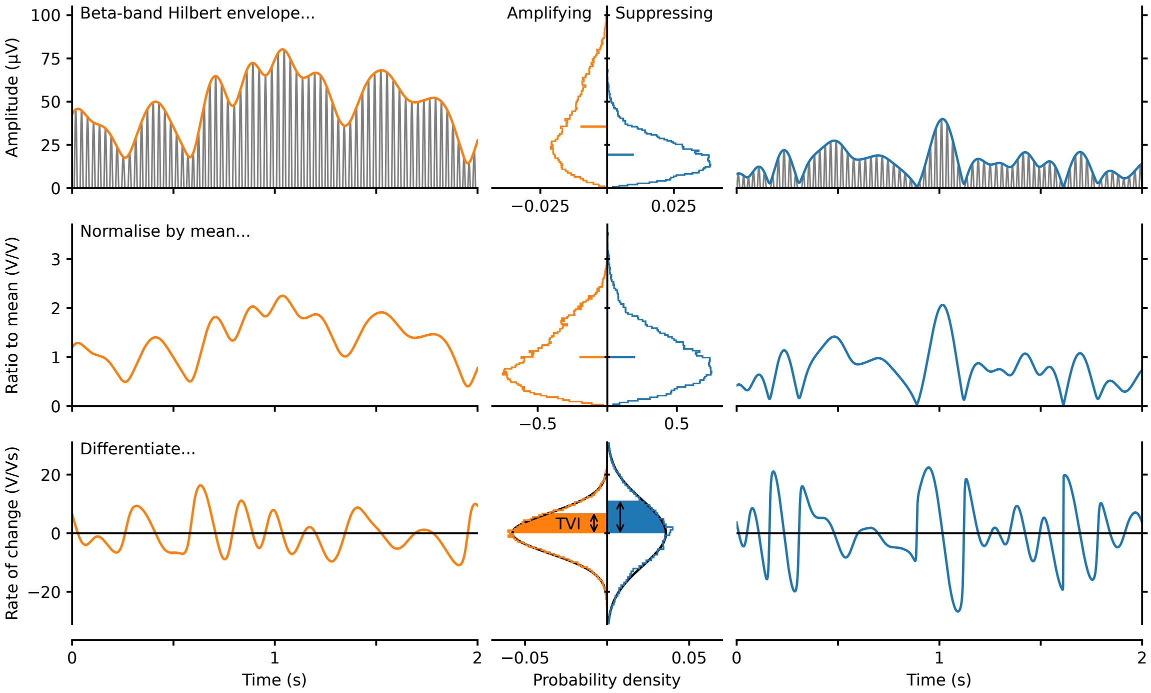

Figure S5. Temporal variation index (TVI).
Related to Figure 3.
We developed the TVI to provide a measure of beta-band amplitude stability over time independent of differences in mean amplitude. The left (orange) shows the calculation performed on a two-second portion from a recording with stimulation targeted to an amplifying phase whereas the right (blue) is from a suppressing recording. The resulting distributions from the full recording in each case are shown in the middle column. The Hilbert amplitude envelope (top) was calculated from the beta-band filtered signal (grey) resulting in different mean amplitudes. Normalising by the mean resulted in similar amplitude distributions (middle). The standard deviation of these distributions is the coefficient of variation (CV). Here, this provides a normalised measure of variation in amplitude, not variation in time (as is the case when the same measure is applied to time intervals such as neuronal action potential firing). To provide a measure of the fluctuations in amplitude over time we calculated the rate of change (differential) of the amplitude envelope before calculating the standard deviation (bottom). Hence, we defined the TVI as the standard deviation of the derivative of the Hilbert amplitude envelope divided by the envelope mean. It is equivalent and slightly computationally advantageous when calculating the TVI to divide by the mean after differentiation and calculation of the standard deviation. As shown, the derivative results in normally distributed values centred on zero allowing the standard deviation to be fully descriptive of the resulting variation. Here, amplifying stimulation produced a smaller TVI than suppressing stimulation indicating a more stable (less variable) oscillation amplitude.

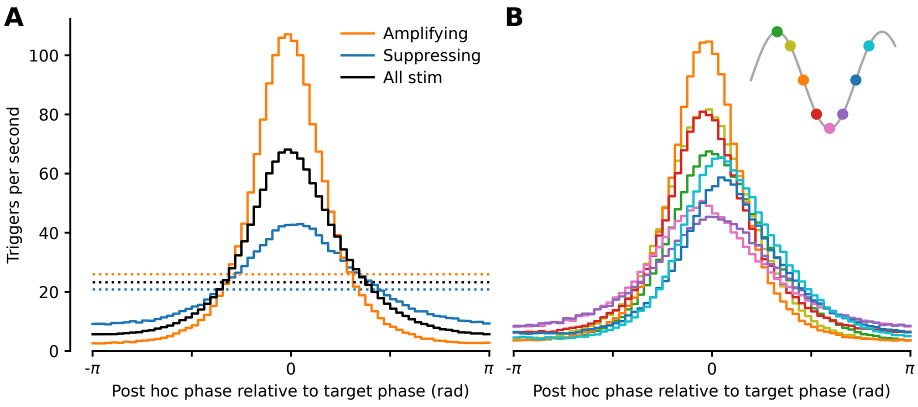

Figure S6. Stimulation rate and accuracy reflected the direction of beta power modulation.
Related to Figure 3.
(A) Trigger accuracy histogram of ECoG phase calculated post hoc plotted relative to target phase. Stimulation targeted to the most amplifying phase (orange) produced a higher rate of stimulation on the correct phase (0 rad) and a higher mean stimulation rate (dotted lines) compared to the mean across all target phases (black). Stimulation targeted to the most suppressing phase (blue) led to reduced accuracy and a lower mean stimulation rate (dotted lines) reflecting the systems success in suppressing the oscillation.
(B) Mean trigger accuracy histograms for each target phase plotted relative to target phase with inset showing the target phase of each trace.

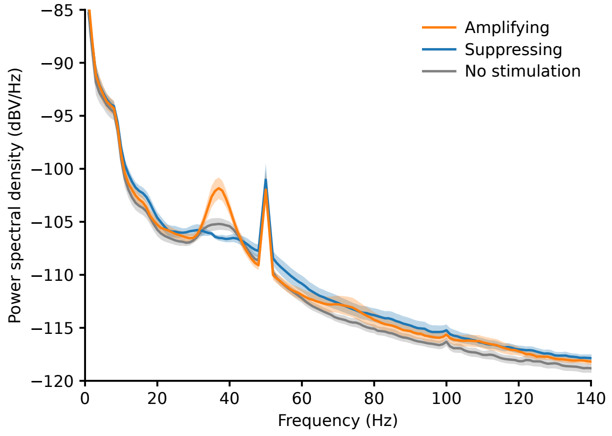

Figure S7. Power spectra from linear track gate analysis experiments.
Related to Figure 4.
Amplifying, suppressing and no stimulation power spectral density plots (mean ± SEM, n = 7 rats). The spike at 50 Hz is due to interference from the mains power.
